# Effects of a Cycling‐Based Aerobic Exercise Program Versus a Resistance and Neuromuscular Exercise Program on Exercise‐Induced Hypoalgesia in Knee Osteoarthritis: A Study Protocol for a Randomized Clinical Trial

**DOI:** 10.1002/pri.70341

**Published:** 2026-09-05

**Authors:** Bruno Ruocco Verengue, Lisa C. Carlesso, Barbara Greco Miura, Aron Charles Barbosa da Silva, Patrícia Gabrielle dos Santos, Almir Vieira Dibai‐Filho, Cid André Fidelis‐de‐Paula‐Gomes

**Affiliations:** ^1^ Postgraduate Program in Rehabilitation Sciences Nove de Julho University São Paulo Brazil; ^2^ School of Rehabilitation Science McMaster University Hamilton Ontario Canada; ^3^ Postgraduate Program in Physical Education Universidade Federal do Maranhão São Luís Maranhão Brazil

**Keywords:** exercise therapy, hypoalgesia, knee osteoarthritis, pain threshold

## Abstract

**Background and Purpose:**

Exercise therapy is a core treatment for knee osteoarthritis (KOA), but the effects of different exercise modalities on endogenous pain modulation remain unclear. This trial will compare a cycling‐based aerobic exercise program with a resistance and neuromuscular exercise program on exercise‐induced hypoalgesia (EIH) in people with KOA and altered baseline EIH.

**Methods:**

This randomized, two‐arm, controlled, single‐blind trial will include 90 participants aged 40–75 years with symptomatic KOA, knee pain intensity of at least 3 points on the Numerical Pain Rating Scale, and altered baseline EIH. Participants will be randomized 1:1 to the Cycling‐Based Aerobic Exercise Group (CAEG) or the Resistance and Neuromuscular Exercise Group (RNEG). Both groups will receive 30 supervised sessions over 10 weeks. The primary treatment effect will be the adjusted mean between‐group difference in within‐session change in pressure pain threshold at the most symptomatic knee, averaged across weeks 1, 4, 7, and 10. Secondary outcomes include EIH at other sites, conditioned pain modulation, pain, self‐efficacy, function, performance, strength, global perceived effect, adherence, and enjoyment.

**Results:**

This trial will evaluate whether the two exercise programs differ in their capacity to elicit EIH in KOA.

**Discussion:**

This trial may clarify whether different exercise modalities produce distinct effects on acute endogenous pain modulation in KOA and may support a more precise exercise prescription for pain management.

**Trial Registration:**

ClinicalTrials.gov, identifier NTC07302204

## 1 Introduction

Knee osteoarthritis (KOA) is a prevalent chronic musculoskeletal condition marked by pain, impaired physical function, and reduced quality of life. Osteoarthritis is a heterogeneous, whole‐joint and whole‐person condition influenced by structural, inflammatory, metabolic, biomechanical, and pain‐related mechanisms (Tang et al. 2025; Collins et al. 2026). Therefore, nonsurgical interventions that improve symptoms and support self‐management remain central to rehabilitation (Tang et al. 2025).

Exercise is recommended as a core conservative intervention for KOA, but evidence remains heterogeneous across modalities, doses, progression, follow‐up duration, and outcomes (Gray et al. 2024; Kitagawa et al. 2025; Rausch Osthoff et al. 2026; Schleimer et al. 2026; Viderman et al. 2026). Aerobic, strengthening, neuromotor, mixed, and mind‐body exercises can improve pain, function, gait performance, quality of life, and physical performance (Yan et al. 2025). These findings do not justify prioritizing a single modality for all individuals. Direct comparisons are needed to determine whether relevant exercise programs yield distinct clinical and mechanistic effects.

A comparison of cycling‐based aerobic exercise with resistance and neuromuscular exercise is clinically relevant. Cycling is a low‐impact modality that can be standardized and progressed using perceived exertion, pain, and safety parameters, and may target systemic mechanisms involved in pain modulation (Song et al. 2023; Anderheide et al. 2025). Resistance and neuromuscular exercise are widely used in KOA rehabilitation to improve strength, neuromotor control, function, and movement confidence (Yan et al. 2025). Both approaches may induce exercise‐induced hypoalgesia (EIH), but responses remain variable (Fingleton et al. 2017; Anderheide et al. 2025). It remains unknown whether these programs differ in their capacity to restore EIH in people with KOA who have altered EIH at baseline.

EIH is a short‐term reduction in pain or pain sensitivity following acute exercise and is commonly assessed by pre‐ to post‐exercise changes in pressure pain threshold (PPT) (Wewege and Jones 2021; Song et al. 2023; Toomey et al. 2025). In KOA, responses range from increased PPT to no change or increased pain sensitivity after exercise (Fingleton et al. 2017; Anderheide et al. 2025; Lee et al. 2025; Toomey et al. 2025). This variability is clinically relevant because impaired EIH may influence exercise tolerance, adherence, and clinical response (Song et al. 2023; Lee et al. 2025; Toomey et al. 2025). PPT was selected as the primary mechanistic measure because it quantifies mechanical pain sensitivity and captures the within‐session response used to define EIH. Conditioned pain modulation (CPM) will be assessed as a secondary outcome because it provides complementary information about descending inhibitory function, although CPM and EIH are not interchangeable constructs (Fingleton et al. 2017; Lee et al. 2025).

Clinical and functional secondary outcomes will assess whether mechanistic changes are accompanied by changes in pain, self‐efficacy, knee‐related and patient‐specific function, physical performance, strength, perceived global effect, adherence, and enjoyment. The primary aim was to compare the effects of cycling‐based aerobic exercise and resistance and neuromuscular exercise on knee EIH in individuals with KOA and altered baseline EIH. We hypothesize that the two programs will differ in their capacity to restore knee EIH, as reflected by between‐group differences in within‐session changes in knee PPT over the 10‐week intervention.

## 2 Methods

### 2.1 Study Design and Recruitment

This randomized, two‐arm, controlled, single‐blind clinical trial will be conducted at the University Clinics of the Nove de Julho University in São Paulo, Brazil. The protocol adheres to the updated CONSORT 2025 and SPIRIT 2025 recommendations and uses the Template for Intervention Description and Replication (TIDieR) checklist to support transparent reporting of the intervention (Hoffmann et al. 2014; Chan et al. 2025; Hopewell et al. 2025) (Supporting Information S1: Table S1).

Recruitment will occur through posters, leaflets, educational talks, and referrals from clinic staff. The target is approximately five eligible participants per month, though this may vary with screening availability and changes in baseline EIH. Screening will include initial contact, informed consent, clinical eligibility assessment, baseline assessment, and randomization only after baseline procedures.

This study was approved by the Research Ethics Committee of Nove de Julho University (No. 93560425.7.0000.5511) and prospectively registered on ClinicalTrials.gov (identifier NTC07302204). Planned recruitment and follow‐up will run from April 2026 to December 2029, aligned with the doctoral timeline and trial logistics. Recruitment may end earlier if the target sample is reached. The same research setting, recruitment sources, and core research team will be maintained throughout the study, whenever possible, to minimize protocol drift and ensure consistency in eligibility assessment, outcome measurement, and intervention delivery.

Recruitment and eligibility assessment will be conducted by two trained physiotherapists using a standardized checklist (Fitzgerald et al. 2015). Before recruitment, the blinded outcome assessor will complete standardized PPT training and a test‐retest reliability assessment in 15 people with KOA. Reliability of the mean of three readings will be estimated using a two‐way mixed‐effects, absolute‐agreement ICC, the standard error of measurement (SEM), and the 95% minimal detectable change (MDC95). An ICC ≥ 0.80 will be required; otherwise, retraining and reassessment will be performed (Stausholm et al. 2023).

Inclusion criteria were men and women aged 40 to 75 years with a prior medical diagnosis of symptomatic KOA, referral for rehabilitation, symptoms for at least 3 months, self‐reported knee pain for more than 3 months, and knee pain intensity of ≥ 3 on the Numerical Pain Rating Scale (NPRS). This age range targets adults commonly affected by symptomatic KOA while minimizing inclusion of younger individuals with secondary knee pain or older individuals with exercise‐limiting comorbidities. KOA will be clinically confirmed using the American College of Rheumatology criteria, defined as knee pain plus at least three of the following: age > 50 years, morning stiffness < 30 minutes, crepitus, bony tenderness, bony enlargement, and absence of palpable warmth (Altman et al. 1986; Zhang et al. 2010).

Participants must also demonstrate altered baseline EIH. After 10 minutes of rest, PPT will be measured using a digital algometer (Force Ten FDX‐25, Wagner Instruments, Greenwich, USA) with a 1 cm^2^ probe, applying pressure at 0.5 kgf/s until the sensation first becomes painful and the participant says “stop” (Rolke et al. 2006; Anderheide et al. 2025).

The PPT will be assessed by the same evaluator at the medial knee compartment of the most symptomatic knee and bilaterally at the upper trapezius. The knee site will be the joint line, 2–3 cm medial to the inferomedial patellar angle, with the participant supine. The remote site will be 10 cm medial to the acromion at C7, with the participant seated. Three measurements will be obtained per site at 30‐second intervals. The mean value will be used, and skin markings will improve reproducibility.

After the baseline PPT assessment, participants will perform an isometric quadriceps contraction of the most symptomatic lower limb in the supine position until voluntary failure or for a maximum of 5 minutes. Total contraction time, knee pain intensity, and perceived exertion will be recorded (Toomey et al. 2025). PPT will be reassessed immediately after exercise at the same marked sites and in the same order.

EIH will be calculated as ΔPPT = post‐exercise PPT minus pre‐exercise PPT, with percentage change also reported (Toomey et al. 2025). Preserved EIH will be defined as ΔPPT > 0 and altered EIH as ΔPPT ≤ 0. Because no validated threshold exists, this is an operational eligibility criterion rather than a diagnostic cutoff. Averaging three readings, standardized sites, skin marking, and the same assessor will reduce measurement error. Baseline ΔPPT will remain continuous in all analyses, and a sensitivity analysis will exclude participants with eligibility ΔPPT values from −MDC95 to 0.

Safety‐related exclusion criteria include cardiovascular or cardiopulmonary contraindications to exercise, deep vein thrombosis or thrombophlebitis, infected wounds or osteomyelitis of the knee, severe osteoporosis or high fracture risk, recent knee trauma, prior arthroplasty of any lower‐limb joint, or neurological disorders that compromise safe exercise participation.

Exclusions include hip symptoms as the primary pain source, fibromyalgia, active inflammatory joint disease, active cancer or cancer‐related pain, clinically significant lower‐limb sensory impairment, cognitive impairment that limits consent or procedure comprehension, use of assistive walking devices, or any other condition deemed to compromise eligibility assessment, intervention safety, adherence, or outcome validity (Fitzgerald et al. 2015).

### 2.2 Sample Size

The sample size was calculated for the prespecified primary estimand, the adjusted mean between‐group difference in knee ΔPPT averaged across A1, A4, A7, and A10. The target difference of 0.30 kgf/cm^2^ and the standard deviation of 0.70 kgf/cm^2^ were taken from the within‐session knee PPT change in the feasibility trial of Anderheide et al. (2025). Under compound symmetry with four measurements and a within‐participant correlation of 0.60, the standard deviation of the participant‐level mean is 0.586 kgf/cm^2^ [0.70 × √((1 + 3 × 0.60)/4)]. Assuming a correlation of 0.60 between baseline PPT and that mean, covariate adjustment gives a residual standard deviation of 0.469 kgf/cm^2^ [0.586 × √(1 − 0.60^2^)].

With a two‐sided *α* of 0.05% and 80% power, *n* = 2(z_1_−α/_2_ + z_1_−β)^2^σ^2^/δ^2^ gives 38.3, that is 39 evaluable participants per group (R 4.4.1). Allowing for approximately 15% attrition, 45 participants per group will be recruited, a total of 90, retaining at least 38 evaluable participants per group and power close to 80%. The estimate depends on the assumed baseline correlation: 55 and 60 participants per group would be needed for correlations of 0.30 and 0.

Compound symmetry is a planning assumption, since the analysis will select the covariance structure empirically. Because no established minimal clinically important difference exists for EIH assessed by PPT in KOA, 0.30 kgf/cm^2^ is an a priori planning target rather than a clinical threshold.

### 2.3 Allocation, Randomization, and Blinding

After eligibility confirmation, informed consent, and baseline assessment, 90 participants will be randomized 1:1 to the CAEG or RNEG. An independent researcher not involved in recruitment, assessment, or intervention delivery will generate the allocation sequence before recruitment using permuted blocks of variable sizes (4, 6, and 8). Allocation codes will be placed in sequentially numbered, opaque, identical, and tamper‐proof sealed envelopes. Recruitment physiotherapists will not have access to the sequence, and each envelope will be opened only after the baseline assessment.

Participants and treating physiotherapists cannot be blinded because of the nature of the interventions. Outcome assessors and the statistician will remain blinded until the database is locked. Participants will be instructed not to disclose group allocation or treatment procedures to the assessor or other participants.

If a session is missed, the responsible researcher will contact the participant within 72 hours, making up to three attempts by telephone, email, or text message. Make‐up sessions will be scheduled within the same week whenever possible.

### 2.4 Interventions

The 10‐week treatment duration was selected to provide 30 supervised sessions, a dose that is feasible in clinical practice and consistent with exercise‐based KOA trials (Fitzgerald et al. 2015; Kabiri et al. 2018; Øiestad et al. 2023). Both protocols will be delivered individually by trained physiotherapists using written manuals. Treatment fidelity will be supported by pretrial competency assessment against a standardized checklist, session‐specific checklists, and monthly direct observation of one randomly selected session per therapist by a senior researcher. Adherence to required components, progression decisions, adverse events, and adaptations will be recorded; deviations will prompt feedback and retraining.

#### 2.4.1 Cycling‐Based Aerobic Exercise Group

CAEG sessions will be conducted three times per week for 10 weeks on a horizontal stationary electromagnetic bicycle (Kikos, KR5.6). Seat position will ensure comfort, alignment, and a safe knee range of motion of approximately 25°–35° of flexion at the distal pedal position. Cadence will be maintained at 60–80 rpm during training and at 50–60 rpm during warm‐up and cool‐down. Workload will follow the target effort zone in Supporting Information S1: Table S2 (Thompson et al. 2013; Øiestad et al. 2023; Anderheide et al. 2025; Whitfield and Tomlinson 2025). Participants will maintain usual physical activity and avoid new therapies or exercise programs during the intervention.

Each session will include a 5‐minute warm‐up, a progressive training phase, and a 5‐minute cool‐down. Intensity will be prescribed and monitored using the Borg 6–20 Rating of Perceived Exertion (RPE), with RPE recorded at the 3rd minute, every 5 minutes thereafter, at session end, and after any workload adjustment. Heart rate, blood pressure, peripheral oxygen saturation, and NPRS pain intensity will also be recorded before, during, and after exercise.

Training will be adjusted or interrupted if intolerance symptoms, abnormal cardiovascular responses, SpO_2_ < 88–90%, or NPRS ≥ 7 occur during exercise. Progression will occur when, for two consecutive sessions, pain remains ≤ 5 during and immediately after exercise, returns to the pre‐session level by the following day, and RPE remains within the target zone, consistent with the NEMEX pain‐monitoring approach (Ageberg et al. 2010). Recorded variables will include duration, workload, cadence, distance or energy, heart rate, RPE, NPRS, adverse events, and protocol adaptations.

#### 2.4.2 Resistance and Neuromuscular Exercise Group

The RNEG includes warm‐ups, resistance exercises, and neuromuscular balance training (Weng et al. 2009; Ageberg et al. 2010; Bennell et al. 2014; Li et al. 2016; Cheung et al. 2017; Torstensen et al. 2018; Anderson et al. 2019; Benner et al. 2019; Hislop et al. 2020). The program will be delivered three times per week for 10 weeks, with at least 24 hours between sessions, and will be divided into weeks 1–5 and weeks 6–10, as detailed in Supporting Information S1: Tables S3 and S4. Sessions will last approximately 60 minutes. Exercises will be performed in up to three sets of 8–12 repetitions in weeks 1–5 and 8–15 repetitions in weeks 6–10, with 90‐s rest intervals (Kabiri et al. 2018).

Loads will be standardized using the one‐repetition maximum test, with pain ≤ 5 on the NPRS for weighted exercises. Training loads will be set at 40–60% of 1‐RM (Toigo and Boutellier 2006; Rabelo et al. 2014). For elastic bands, resistance will be selected to allow 12 repetitions with NPRS ≤ 5; for bodyweight exercises, holding time will be used as the progression parameter.

Vital signs and pain intensity will be monitored before, during, and after each session. The physiotherapist will provide instructions, ensure safety, and adjust exercise execution. Exercises involving weights, elastic resistance, or contraction duration will be reassessed weekly. Progression will consist of increasing machine load by 5–10%, selecting a higher‐resistance band, or increasing holding time when pain remains ≤ 5 during and immediately after exercise and returns to the pre‐session level by the following day (Ageberg et al. 2010).

### 2.5 Primary Outcome

The primary outcome is EIH in the medial knee compartment of the most symptomatic knee, measured as intra‐session change in PPT (ΔPPT) at A1, A4, A7, and A10. The primary estimand is the adjusted mean between‐group difference in ΔPPT averaged across the four sessions. The group‐by‐session interaction and session‐specific differences will be exploratory. PPT will be measured immediately before and after each selected session, and ΔPPT will be the mean post‐exercise value minus the mean pre‐exercise value in kgf/cm^2^. Positive values indicate EIH, zero indicates no EIH, and negative values indicate a hyperalgesic response. If both knees are equally symptomatic, the target knee will be predefined and maintained throughout the trial.

PPT will be measured using a digital algometer (Force Ten FDX‐25, Wagner Instruments, Greenwich, USA) using a 1 cm^2^ probe. Pressure will be applied at 0.5 kgf/s until the sensation first becomes painful and the participant indicates “stop” (Rolke et al. 2006; Anderheide et al. 2025). Three readings will be obtained at the target knee using the same evaluator, in the same order, with 30‐s intervals. The arithmetic mean will be used for analysis, and skin marking will be used to improve site reproducibility.

The knee PPT site will be the medial compartment at the joint line, 2–3 cm medial to the inferomedial angle of the patella, with the participant supine. Intra‐session EIH will be calculated only at A1, A4, A7, and A10. T0 and T1 PPT assessments will characterize baseline and post‐intervention pressure pain sensitivity, not acute EIH.

### 2.6 Secondary Outcomes

Secondary EIH outcomes are intra‐session ΔPPT at the rectus femoris and upper trapezius at A1, A4, A7, and A10. Rectus femoris PPT will be assessed bilaterally with the participant supine, 15 cm proximal to the base of the patella. Upper trapezius PPT will be assessed bilaterally with the participant seated and 10 cm medial to the acromion at the level of C7. For bilateral sites, the right and left values will be averaged for analysis. Chronic adaptation in PPT will be evaluated at T0 and T1 at the knee, rectus femoris, and trapezius.

CPM will be assessed at T0 and T1 using conventional PPT as the test stimulus and ischemic compression as the conditioning stimulus, consistent with CPM testing recommendations (Yarnitsky et al. 2015). Baseline PPT will be measured on the anterior surface of the right forearm, 7.5 cm from the distal wrist crease, using the same algometer and an application rate of 0.5 kgf/s. PPT algometry demonstrates acceptable to excellent reliability in people with KOA (Stausholm et al. 2023). For the conditioning stimulus, an aneroid sphygmomanometer was positioned on the left arm, 3 cm proximal to the cubital fossa, and inflated to 250 mmHg. If pain is < 5 on the NPRS, participants will perform elbow flexion and extension until pain reaches ≥ 5. PPT will then be reassessed on the right forearm while the conditioning stimulus is maintained. After cuff release, PPT will be reassessed at 30 seconds and 5 minutes. CPM will be calculated as the change in PPT during conditioning relative to baseline PPT, with higher values indicating more efficient modulation.

Pain intensity will be assessed at T0 and T1 using the Brazilian Portuguese Numerical Pain Rating Scale (NPRS), ranging from 0 (“no pain”) to 10 (“worst imaginable pain”), for average pain at rest and after movement over the previous 7 days. A 2‐point change will serve as the reference threshold for clinical interpretation (Farrar et al. 2001; Costa et al. 2008).

Pain self‐efficacy will be assessed at T0 and T1 using the Brazilian Portuguese Pain Self‐Efficacy Questionnaire (PSEQ), a 10‐item scale scored from 0 to 60, with higher scores indicating greater self‐efficacy. An 11‐point change will serve as the reference threshold for clinical interpretation (Sardá et al. 2007; Dubé et al. 2021).

Knee health status will be assessed at T0 and T1 using the Brazilian Portuguese Knee Injury and Osteoarthritis Outcome Score (KOOS), which includes the Pain, Symptoms, Activities of Daily Living, Sport/Recreation, and Knee‐Related Quality of Life subscales. Scores will be transformed into a 0–100 scale, with higher scores indicating better status. A 10‐point change per subscale will serve as the reference threshold (Roos and Lohmander 2003; Almeida et al. 2022; Pathak et al. 2022).

Patient‐specific function will be assessed at T0 and T1 using the Patient‐Specific Functional Scale (PSFS), in which participants identify three KOA‐related activities and rate each on a scale from 0 to 10. The score is the mean of the three activities, and the same activities identified at baseline will be re‐rated at T1. PSFS has strong measurement properties across musculoskeletal conditions, and changes of ≥ 2 points are generally considered clinically meaningful (Moore et al. 2020; Dubé et al. 2021; Pathak et al. 2022).

Lower‐limb functional performance will be assessed at T0 and T1 using the 30‐s sit‐to‐stand test (30STST), recommended by OARSI for KOA. Using a 43 cm armless chair against a wall, participants will keep their arms crossed and complete as many full repetitions as possible in 30 seconds after 1–2 practice repetitions. The score will be the number of full repetitions completed (Dobson et al. 2013; Gill et al. 2022).

Quadriceps strength will be assessed at T0 and T1 using maximal voluntary isometric contraction (MVIC) measured with a handheld dynamometer (Lafayette Manual Muscle System, Model 01163). Participants will sit with their hips at 90° and knees at 60°. The dynamometer will be positioned at the malleolar level and secured with a non‐elastic strap during knee extension. Force will be normalized to body weight (Martin et al. 2006; de Paula Gomes et al. 2018).

Global perceived effect will be assessed at T1 using an 11‐point scale ranging from −5 (“much worse”) to +5 (“completely recovered”), with a 2‐point change serving as the reference threshold for clinical interpretation (Kamper et al. 2009). Adherence will be calculated as attendance (%) = (attended sessions/planned sessions) × 100.

Exercise enjoyment will be assessed at T1 using the Brazilian Portuguese Physical Activity Enjoyment Scale (PACES), an 18‐item, 7‐point bipolar‐response scale measuring perceived enjoyment during exercise. After recoding the required items, higher scores indicate greater enjoyment. The Brazilian Portuguese version demonstrated almost perfect reproducibility, with a minimal detectable change of 23.9 points (Alves et al. 2019).

## 3 Statistical Analysis

Statistical analyses will follow the intention‐to‐treat principle. The primary outcome will be analyzed using a linear mixed model with fixed effects for group, assessment session (A1, A4, A7, and A10), and group‐by‐session interaction; baseline PPT at the corresponding site will be included as a covariate, with a random intercept for participants.

The primary treatment effect will be the adjusted mean between‐group difference in ΔPPT averaged across the four sessions, estimated using marginal means with 95% confidence intervals and *α* = 0.05. The group‐by‐session interaction and session‐specific contrasts will be exploratory. The covariance structure will be selected using the Akaike Information Criterion from prespecified compound‐symmetry, autoregressive, and unstructured structures.

Continuous secondary outcomes measured at T0 and T1, including KOOS, 30STST, NPRS, CPM, PSEQ, PSFS, and MVIC, will be analyzed using linear mixed models with fixed effects for group, time, and group‐by‐time interaction and a random participant intercept. T1‐only outcomes, including PACES and GPE, will be analyzed using regression models with group as the predictor and an appropriate link function if distributional assumptions are not met.

The primary outcome will be the sole confirmatory outcome because the trial is powered only for it. All secondary outcomes are prespecified but will be interpreted as exploratory and supportive, with emphasis on effect sizes and 95% confidence intervals rather than on confirmatory hypothesis testing.

Clinically important thresholds, when available, will be used only as reference values to support interpretation and not as confirmatory decision rules.

Mixed models will use all available data under the missing‐at‐random assumption. For outcomes not accommodated by mixed models, multiple imputation by chained equations will be used when more than 5% of observations are missing, incorporating group, available baseline and outcome values, adherence, and predictors of missingness. Complete‐case analyses will be sensitivity analyses only. Adherence will be summarized as attendance percentage; a per‐protocol sensitivity analysis will define adequate adherence as ≥ 80%. Protocol deviations, adverse events, and adaptations will be summarized descriptively. Analyses will be conducted in *R*.

## 4 Discussion

This randomized clinical trial will compare cycling‐based aerobic exercise with resistance and neuromuscular training in people with KOA and altered baseline EIH. Repeated within‐session knee PPT assessments will determine whether the two active programs produce distinct effects on acute pain modulation over 10 weeks (Anderheide et al. 2025). Both approaches are clinically relevant, yet their comparative effects on endogenous pain modulation remain unclear. Concealed allocation, blinded assessment and analysis, standardized protocols, treatment‐fidelity procedures, and intention‐to‐treat analysis should reduce bias. The pragmatic sample size and exploratory status of secondary outcomes should be considered when interpreting findings.

Restricting eligibility to people with altered baseline EIH addresses the mechanistic question but limits generalizability to the broader symptomatic KOA population, particularly individuals with preserved EIH. The operational ΔPPT ≤ 0 criterion may also classify changes within measurement error as altered EIH. Continuous analyses, assessor reliability estimates, and the MDC95‐based sensitivity analysis will contextualize this uncertainty.

## 5 Implications for Physiotherapy Practice

This trial may inform physiotherapy practice by clarifying whether cycling‐based aerobic exercise, resistance training, and neuromuscular training elicit distinct EIH responses in people with KOA. The findings may help clinicians integrate pain modulation, function, safety, adherence, and enjoyment when selecting exercise programs for individuals with chronic knee pain, rather than assuming that one modality should be prioritized for all patients.

## Author Contributions

**Bruno Ruocco Verengue:** writing – review and editing, writing – original draft, supervision, software, methodology, formal analysis, conceptualization. **Lisa C. Carlesso:** supervision, project administration, methodology. **Barbara Greco Miura:** investigation and data curation. **Aron Charles Barbosa da Silva:** investigation and data curation. **Patrícia Gabrielle dos Santos:** investigation, data curation. **Almir Vieira Dibai‐Filho:** methodology, supervision. **Cid André Fidelis‐de‐Paula‐Gomes:** writing – review and editing, writing – original draft, supervision, project administration, methodology.

## Funding

The authors have nothing to report.

## Ethics Statement

Ethical approval was obtained from the research ethics committee of the Nove de Julho University, São Paulo (No. 93560425.7.0000.5511) and was prospectively registered on the Clinical Trials platform under the identifier NTC07302204.

## Conflicts of Interest

The authors declare no conflicts of interest.

## Trial Status

Planned recruitment and follow‐up will run from April 2026 to December 2029, aligned with the doctoral timeline and trial logistics.

## Supporting information


Supporting Information S1

## Data Availability

The authors have nothing to report.
