# Supplementary material for: Effects of a Cycling‐Based Aerobic Exercise Program Versus a Resistance and Neuromuscular Exercise Program on Exercise‐Induced Hypoalgesia in Knee Osteoarthritis: A Study Protocol for a Randomized Clinical Trial
Source: Physiother Res Int. 2026 Sep 5;31(4):e70341. doi: 10.1002/pri.70341 (PMC13545987; doi:10.1002/pri.70341)
Supplement: Supplementary file 1 [file PRI-31-e70341-s001.docx]

|  | **Study Periods** | | | | | | |
| --- | --- | --- | --- | --- | --- | --- | --- |
|  | Recruitment | | Post Allocation | | | | Follow-up |
| Times | 0 -T_0_ | T_0_ | A_1_ | A_4_ | A_7_ | A_10_ | T_1_ |
| **PARTICIPATION**: |  |  |  |  |  |  |  |
| Elegibility criteria | X |  |  |  |  |  |  |
| Informed consent | X |  |  |  |  |  |  |
| Random sequence Generation | X |  |  |  |  |  |  |
| Confidentiality allocation |  | X |  |  |  |  |  |
| **INTERVENTIONS:** |  |  |  |  |  |  |  |
| CAEG |  | X |  |  |  |  | X |
| RNEG |  | X |  |  |  |  | X |
| **EVALUATIONS**: |  |  |  |  |  |  |  |
| [PPT] |  | X | X | X | X | X | X |
| [CPM] |  | X |  |  |  |  | X |
| [NPRS] |  | X |  |  |  |  | X |
| [PSEQ] |  | X |  |  |  |  | X |
| [KOOS] |  | X |  |  |  |  | X |
| [PSFS] |  | X |  |  |  |  | X |
| [30STST] |  | X |  |  |  |  | X |
| [MVIC] |  | X |  |  |  |  | X |
| [PACES] |  |  |  |  |  |  | X |
| [GPE] |  |  |  |  |  |  | X |
| Statistical Analysis |  |  |  |  |  |  | X |

Table S1. Schematic diagram of Spirit Statement overall study schedule

T0: pre-intervention, T1: post 10 weeks intervention; A1: first session of week 1, A4: first session of week 4, A7: first session of week 7, A10: first session of week 10; PPT: Pressure pain threshold, CPM: Conditioned pain modulation, NPRS: Numerical Pain Rating Scale, PSEQ: Pain Self-efficacy Questionnaire, KOOS: Knee injury and osteoarthritis outcome score; PSFS: Patient-specific functional scale; 30STST: 30-second sit-to-stand test; MVIC: maximal voluntary isometric contraction, PACES: physical activity enjoyment scale, GPE: Global Perceived Effect Scale, CAEG: Cycling-Based Aerobic Exercise Group, RNEG: Resistance and Neuromuscular Exercise Group. Note: PPT assessments at A1, A4, A7, and A10 refer to intra-session measurements performed immediately before and immediately after the corresponding exercise session to calculate ΔPPT. PPT assessments at T0 and T1 refer to baseline and post-intervention pressure pain sensitivity, not to the intra-session EIH response.

Table S2. Protocol description of Cycling-Based Aerobic Exercise Group over 10 weeks.

| Period | Description |
| --- | --- |
| Weeks 1-2 | - 5-min warm-up (RPE 8-10)  - 15-20 min training phase (RPE 10-11 during the first 2-3 sessions, progressing to 12)  - 5-min cool-down (RPE 8-10) |
| Weeks 3-5 | - 5-min warm-up (RPE 8-10)  - 25-30 min training phase (RPE 12-13)  - 5-min cool-down (RPE 8-10) |
| Weeks 6-8 | - 5-min warm-up (RPE 8-10)  - 30-35 min training phase (RPE 12-13)  - 5-min cool-down (RPE 8-10) |
| Weeks 9-10 | - 5-min warm-up (RPE 8-10) - 35-40 min training phase (RPE 12-13) - 5-min cool-down (RPE 8-10) |

RPE: Rating of perceived exertion.

Table S3. Protocol description of Resistance and Neuromuscular Exercise Group, 1-5 weeks.

| Weeks | Exercise | Description | Characteristics |
| --- | --- | --- | --- |
| Weeks 1-5 | Warm-up | Warm-up performed on a treadmill (Inbramed^®^, Master TOP 18) or stationary bicycle (Matrix^®^, upright) for 10 minutes at low intensity. | Treadmill without incline adjustment, with speed standardized between 2.0 and 4.0 km/h, or cycling on a stationary bicycle (50-70 rpm; resistance levels 1-5). |
|  | Horizontal leg press | Participants are seated with the lumbar region supported against the backrest, feet positioned shoulder-width apart on the platform, knees flexed at 90°, and hands placed on the machine handles (Technogym^®^, Selection 900). Following instructions, participants perform a purely concentric action and return slowly to the starting position before the next repetition. A 1-second pause is maintained between eccentric and concentric phases. An experienced researcher assists in maintaining foot positioning on the platform during both phases and provides verbal encouragement throughout execution. | Three sets of 8-12 repetitions each, with 90-second rest intervals between sets. |
|  | Knee extension machine | Participants are seated on the knee extension machine (Nakagym^®^) with the lumbar region supported against the seat and ankles positioned behind the equipment bar. Seat height is adjusted according to limb length. Initial position at 90° knee flexion and final position at 45° knee flexion. A researcher provides verbal encouragement and assists in maintaining appropriate joint angle and positioning throughout the movement. | Three sets of 8-12 repetitions each, with 90-second rest intervals between sets. |
|  | Sit-to-stand | Participants are seated on a chair with feet parallel and body weight distributed evenly on both legs, initially using light hand support for balance and progressing to no hand support. Participants are instructed to repeatedly perform the sit-to-stand movement. | Three sets of 8-12 repetitions each, with 90-second rest intervals between sets. |
|  | Hip abduction and external rotation at 30° hip flexion (clam exercise) | Participants lie in the side-lying position on a treatment table with feet together and hips and knees flexed to approximately 30°, with an elastic resistance band (Theraband^®^) placed around the knees. A researcher stands behind the participant to stabilize the trunk and pelvis, preventing compensatory movements during the exercise. Participants are instructed to keep the feet together while lifting the upper knee. | Three sets of 8-12 repetitions each, with 90-second rest intervals between sets. |
|  | Bridge exercise | Participants lie in the supine position on a treatment table with knees flexed and an elastic resistance band (Theraband^®^) placed around the knees. Participants perform hip elevation from the table (bridge movement). | Three sets of 8-12 repetitions each, with 90-second rest intervals between sets. |
|  | Weight-bearing stabilization (Trendelenburg exercise) | Participants stand with one lower limb positioned on a step platform (Legacy^®^) while the contralateral limb remains suspended beside it, with hands on hips and trunk/pelvis maintained in a neutral position. Participants are instructed to perform hip adduction attempting to touch the ground with the suspended foot and then return to the starting position without compensatory contralateral pelvic elevation. An ankle weight (Fisic^®^) is positioned around the distal third of the leg. | Three sets of 8-12 repetitions each, with 90-second rest intervals between sets. |

Table S4. Protocol description of Resistance and Neuromuscular Exercise Group, 6-10 weeks.

| Weeks | Exercise | Description | Characteristics |
| --- | --- | --- | --- |
| Weeks 6-10 | Warm-up | Warm-up performed on a treadmill (Inbramed^®^, Master TOP 18) or stationary bicycle (Matrix^®^, upright) for 10 minutes at low intensity. | Treadmill without incline adjustment, with speed standardized between 2.0 and 4.0 km/h, or cycling on a stationary bicycle (50-70 rpm; resistance levels 1-5). |
|  | Horizontal leg press | Participants are seated with the lumbar region supported against the backrest, feet positioned shoulder-width apart on the platform, knees flexed at 90°, and hands placed on the machine handles (Technogym^®^, Selection 900). Following instructions, participants perform a purely concentric action and return slowly to the starting position before the next repetition. A 1-second pause is maintained between eccentric and concentric phases. An experienced researcher assists in maintaining foot positioning on the platform during both phases and provides verbal encouragement throughout execution. | Three sets of 8-15 repetitions each, with 90-second rest intervals between sets. |
|  | Knee extension machine | Participants are seated on the knee extension machine (Nakagym^®^) with the lumbar region supported against the seat and ankles positioned behind the equipment bar. Seat height is adjusted according to limb length. Initial position at 90° knee flexion and final position at 45° knee flexion. An experienced researcher provides verbal encouragement and assists in maintaining appropriate joint angle and positioning throughout the movement. | Three sets of 8-15 repetitions each, with 90-second rest intervals between sets. |
|  | Squat | Participants are seated on a chair with feet parallel and one foot positioned slightly in front of the other, initially using hand support for balance and progressing to no hand support. Participants are instructed to perform the sit-to-stand movement from the chair. | Three sets of 8-15 repetitions each, with 90-second rest intervals between sets. |
|  | Straight leg raise with slight hip extension | Participants are positioned in side-lying with the knee fully extended and the hip in a neutral position. They are instructed to abduct the upper leg until reaching approximately 30°, while maintaining neutral alignment in the transverse plane. The researcher remains positioned behind the participant to prevent pelvic displacement during the exercise. An ankle weight (Fisic^®^) is attached to the distal third of the leg. | Three sets of 8-15 repetitions each, with 90-second rest intervals between sets. |
|  | Weight transfer | Participants stand facing a treatment table while supporting themselves with both upper limbs on the table. The researcher instructs participants to lift one leg, transferring body weight from one leg to the other. Once the participant demonstrates adequate control, support from one hand and subsequently the other is removed. | Three sets of 30-60 seconds, performed with eyes open and closed, with 90-second rest intervals between sets. |
|  | Tandem gait | Participants are instructed to perform tandem gait (heel-to-toe walking, with the heel of the leading foot touching the hallux of the trailing foot) over a 3-meter distance along a marked line on the floor as quickly as possible. | Three sets with 90-second rest intervals between sets. |
|  | Multi-directional step-down | Participants stand on a 10-cm step platform (Legacy^®^) and step down and back up in alternating directions (forward, backward, and lateral), returning to the starting position according to the researcher’s commands. | Three sets of 30-60 seconds each, with 90-second rest intervals between sets. |
